# *Chlamydia* pan-genomic analysis reveals balance between host adaptation and selective pressure to genome reduction

**DOI:** 10.1186/s12864-019-6059-5

**Published:** 2019-09-12

**Authors:** Olga M. Sigalova, Andrei V. Chaplin, Olga O. Bochkareva, Pavel V. Shelyakin, Vsevolod A. Filaretov, Evgeny E. Akkuratov, Valentina Burskaia, Mikhail S. Gelfand

**Affiliations:** 1 Kharkevich Institute for Information Transmission Problems, RAS, Moscow, Russia; 2 current address: European Molecular Biology Laboratory, Heidelberg, Germany; 3 Microbiology and Virology Department, Pirogov Russian National Research Medical University, Moscow, Russia; 4 current address: Institute of Science and Technology Austria, Klosterneuburg, Austria; 5 Center for Life Sciences, Skolkovo Institute of Science and Technology, Moscow, Russia; 6 Vavilov Institute of General Genetics, RAS, Moscow, Russia; 7 Institute of Translational Biomedicine, St. Petersburg State University, St. Petersburg, Russia; 8 current address: Science for Life Laboratory, Department of Applied Physics, Royal Institute of Technology, Stockholm, Sweden; 9 Faculty of Computer Science, Higher School of Economics, Moscow, Russia

**Keywords:** *Chlamydia*, Intracellular pathogens, Pan-genome, Genome evolution, Comparative genomics, PmpG

## Abstract

**Background:**

*Chlamydia* are ancient intracellular pathogens with reduced, though strikingly conserved genome. Despite their parasitic lifestyle and isolated intracellular environment, these bacteria managed to avoid accumulation of deleterious mutations leading to subsequent genome degradation characteristic for many parasitic bacteria.

**Results:**

We report pan-genomic analysis of sixteen species from genus *Chlamydia* including identification and functional annotation of orthologous genes, and characterization of gene gains, losses, and rearrangements. We demonstrate the overall genome stability of these bacteria as indicated by a large fraction of common genes with conserved genomic locations. On the other hand, extreme evolvability is confined to several paralogous gene families such as polymorphic membrane proteins and phospholipase D, and likely is caused by the pressure from the host immune system.

**Conclusions:**

This combination of a large, conserved core genome and a small, evolvable periphery likely reflect the balance between the selective pressure towards genome reduction and the need to adapt to escape from the host immunity.

**Electronic supplementary material:**

The online version of this article (10.1186/s12864-019-6059-5) contains supplementary material, which is available to authorized users.

## Background

Bacteria of genus *Chlamydia* are intracellular pathogens of high medical significance. *Chlamydia trachomatis* are important agents of sexually transmitted disease as well as the main cause of preventable blindness in developing countries, and *Chlamydia pneumoniae* is one of the major causes of pneumonia worldwide. Other chlamydial species infect a wide range of animals, and some of them (in particular, *Chlamydia psittaci* and *Chlamydia abortus*) can cause life-threatening diseases if transmitted to humans [1–6]. No vaccines exist against human chlamydial strains, and the number of cases does not decrease with better examination schemas [7, 8]. The recurrence rate is high, and persistent chlamydial infections are associated with higher risk of atherosclerosis, reactive arthritis, and oncogenic effects [2, 7, 9, 10].

*Chlamydia* have a complex biphasic lifecycle [11], which involves changes in DNA compaction [12], metabolism [13], and temporal expression of early, middle, and late genes [14]. Extracellular forms of *Chlamydia* (*elementary bodies*) attach to host cells and initiate endocytosis, yielding formation of a membrane-bound compartment termed the inclusion. Once inside the inclusion, elementary bodies differentiate into a larger intracellular form (*reticulate bodies*), engaging in complex host-pathogen interactions. In particular, these bacteria reorganize vesicular transport, prevent apoptosis, slow down the host cell cycle, and suppress inflammatory immune response by damping nuclear factor B transcription; for a review see [1]. The chlamydial inclusion membrane has been referred to as a pathogen-specified parasitic organelle [15].

As summarized in [16], a typical bacterial genome is shaped by the dynamic interaction of six major evolutionary forces directed towards either genome reduction or complexification. Genome streamlining (1) and degradation (2) both lead to genome contraction, though the underlying evolutionary mechanisms are different. Genome streamlining results from strong positive selection pressure to remove non-essential genes. Genome degradation implies gene loss under weak or neutral selection, which is typically manifested by high numbers of pseudogenes and integrated selfish elements. Genome streamlining is characteristic of highly abundant and evolutionary successful organisms, whereas genome degradation has been reported for some parasitic and symbiotic microorganisms which have small effective population sizes due to their lifestyle [16, 17]. As counteracting forces, genome reduction is contained by genome complexification and innovation via gene duplications (3), operon shuffling (4), horizontal gene transfer (5), or propagation of mobile elements (6). And although all these forces might act simultaneously, their contribution to shaping individual prokaryotic genomes is strikingly different, reflecting the ecological niche and the lifestyle of a microorganism.

As a consequence of their obligatory intracellular lifestyle, *Chlamydia* have reduced genomes of about 1 Mb and 850–1100 genes. At most 14 transcription factors (TFs) have been predicted to regulate gene expression [18]. However, as opposed to many other pathogens with reduced genomes [17], this apparent simplification likely resulted from genome streamlining rather than degradation. In particular, genomes of all chlamydial species have a low number of pseudogenes [2, 19]. The gene order is highly conserved everywhere outside the *plasticity zone*, a genomic region of about 81 kB around the replication terminus [20]. Similarly, the gene content is conserved, with the majority of genes being shared with other representatives of the phylum [21]. Multiple studies shown the genome-wide homologous recombination among *Chlamydia* species, which may prevent accumulation of deleterious mutations [22–24]. Finally, genomes of *Chlamydia* spp. are generally free of disruptive mobile elements, with the exception of the IS-associated tetracycline-resistance genomic island in *C. suis* and the remnants of IS-like elements and prophages in some genomes [2, 25–27].

Furthermore, the reduced genome of *Chlamydia* allows for significant phenotypic variation with sixteen currently recognized species (Table 1) having a broad range of host specificities and tissue tropisms [25–27]. In particular, *C. trachomatis* are exclusively human pathogens causing trachoma (serovars A-C), lymphogranuloma venereum (LGV, serovars L1-L3), and epithelial urogenital infections (serovars D-K). *C. pneumoniae* is one of the most common causes of respiratory infections in humans, also able to infect animals, e.g. horses, marsupials, and frogs. Other species infect a broad range of animals including mice, guinea pigs, birds, cattle, sheep, swine, horses, cats, koalas, frogs, and snakes, causing various diseases, which in some cases can be transmitted to humans (for a review see [2]). The source of this phenotypic diversity is limited, and most differences in the host specificity and tissue tropism have been attributed to several highly variable gene families including cytotoxin [28], polymorphic outer membrane proteins [29], inclusion membrane proteins [30], and phospholipase D enzymes [31], as well as several metabolic pathways, such as tryptophan [32] and biotin [2] biosynthesis.

**Table 1:** Summary of analyzed genomes. *Waddlia chondrophila* is the only bacterium outside genus *Chlamydia*, it was used as an outgroup to construct the rooted phylogenetic tree of the genus

Species	Number of genomes	Number of complete genomes	Median genome size, mB	Median number of CDS
*Chlamydia trachomatis*	110	95	1.04	904
*Chlamydia suis*	30	11	1.09	924
*Chlamydia abortus*	27	7	1.16	982
*Chlamydia psittaci*	25	20	1.17	977
*Chlamydia pneumoniae*	12	12	1.23	1046
*Chlamydia pecorum*	10	6	1.11	950
*Chlamydia muridarum*	3	2	1.07	905
*Chlamydia gallinacea*	2	2	1.06	907
*Chlamydia avium*	1	1	1.04	895
*Chlamydia caviae*	1	1	1.17	981
*Candidatus Chlamydia corallus*	1	0	1.20	1005
*Chlamydia felis*	1	1	1.17	981
*Chlamydia ibidis*	1	0	1.15	955
*Chlamydia poikilothermis*	1	1	1.16	972
*Chlamydia sanzinia*	1	1	1.11	932
*Chlamydia serpentis*	1	1	1.20	992
*Waddlia chondrophila*	1	1	2.12	1839

The pan-genome approach [33] is a comparative genomic technique which involves analysis of conservation and evolution of individual gene families from a clade of microorganisms. To construct the pan-genome, all genes from a group of closely related prokaryotic genomes are pooled together and then clustered into orthologous groups (OGs) by sequence similarity. OGs are then classified into universally conserved (“core”), non-universal (“periphery”), and unique (“singletons”) gene fractions. The structure of the pan-genome provides a robust description of the phylogenetic structure within a prokaryotic group [34, 35] as well as the lifestyle of microorganisms from the group [36]. Due to the high medical relevance of *Chlamydia*, the research on their genomes has greatly expanded over the last decade. Previous studies include pan-genomic analysis of phylum *Chlamydiae* [21] and order *Chlamydiales* [37], as well as multiple comparative studies of individual chlamydial species or gene families.

In this study, we provide a comprehensive pan-genomic analysis of 227 strains from 16 species of genus *Chlamydia* and assess the contribution of genome reduction and complexification processes in shaping the chlamydial genome. The paper is organized as follows. Firstly, we identify and functionally characterize the conserved and variable components of the chlamydial pan-genome. Next, we focus on the interplay between the genome complexification and streamlining via the analysis of gene losses, pseudogenes, genomic rearrangements, and expansion of paralogous gene families. Finally, we provide a case-study of the Polymorphic membrane protein G (PmpG) gene family representing an interesting combination of extensive paralogisation, phase and antigen variation, and pseudogenisation.

## Results

### Pan-genome of genus *Chlamydia*: large core, small periphery, and tiny fraction of singletons

Here, we provide comparative genomic analysis of 16 currently recognized, or candidate, species from genus *Chlamydia* (Table 1). After quality filtering, the dataset comprised 161 complete and 66 draft genomes assembled into up to 10 contigs (Additional file 1: Table S1). The pan-genome (Additional file 2: Table S2 and Additional file 3: Table S3) was constructed by pooling together transcript sequences of all genes and then clustering them into orthologous groups (OGs) using orthoMCL [38]. To avoid biases arising from differences in gene prediction algorithms, genes in all selected genomes were re-annotated de novo using the RAST pipeline [39] prior to orthoMCL clustering. In addition, since RAST predicted multiple putative short open reading frames which might be false positives, OGs comprised exclusively of hypothetical proteins with average length below 50 amino acids were excluded from subsequent analysis. This resulted in removal of 1288 OGs, most of which were present in a small number of genomes (Additional file 4: Figure S1).

**Fig. 1:**
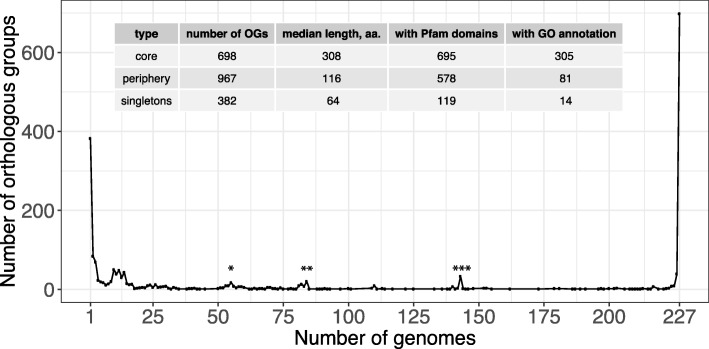
Distribution of Orthologous Groups (OGs) by the number of *Chlamydia* genomes that have them (the U-curve). The left and right peaks of the curve correspond to unique (singletons) and universally conserved (core) genes, respectively. The rest of the OGs form the periphery. Internal peaks are mainly formed by OGs specific to individual species or monophyletic groups of species. In particular, peak at 55 genomes (*) corresponds to genes specific to the clade comprised of *C. abortus*, *C. psittaci*, *C. felis*, *C. caviae*, and *C. poikilothermis*. Peaks at 143 (***) and 84 genomes (**), respectively, are formed by orthologous groups specific to two clades previously assigned to genera *Chlamydia* (*C. trachomatis*, *C. muridarum*, and *C. suis*) and *Chlamydophila* (all other species). The statistics of the pan-genome components (core, periphery, and singletons) is shown in the inset table. It includes the number of OGs in the component, the median protein length, the number of OGs containing genes that have known conserved protein domains (according to Pfam-A), and the number of OGs containing genes with Gene Ontology (GO) annotation

The resulting pan-genome (Fig. 1) consisted of 2047 orthologous groups, out of which 698 were universally conserved in all 227 genomes of genus *Chlamydia* (core). 967 OGs were present in some but not all genomes (periphery), and only 382 proteins were present in a single genome (singletons). Intermediate peaks in Fig. 1 correspond to species-specific OGs (Additional file 5: Table S4) and genes unique to certain monophyletic groups of species. In particular, two peaks of 33 and 19 OGs, at 143 and 84 genomes, respectively, are formed by orthologous groups specific to two clades previously assigned to genera *Chlamydia* (*C. trachomatis*, *C. muridarum*, and *C. suis*) and *Chlamydophila* (all other species), a division proposed in [40] but not generally accepted by the research community [41]. An additional peak of 17 OGs at 55 genomes corresponds to genes specific to the clade comprised of *C. abortus*, *C. psittaci*, *C. felis*, *C. caviae*, and *C. poikilothermis*. Pan-genomes of individual species yield plots similar to that of the genus (Additional file 6: Figure S2), but without intermediate peaks.

Overall, out of about 910 protein-coding genes in an average chlamydial genome, 75% were universally conserved through the whole genus. The share of singletons ranged from 6% in *Chlamydia ibidis* represented by one strain in our dataset to less than 1% in *Chlamydia trachomatis* with 110 strains available. Of note, the pan-genome structure is not distorted by the inclusion of draft genomes into the analyzed dataset (Additional file 7: Figure S3). The number of orthologous groups universally present in the whole genus increased by only 10 OGs after removing 66 draft genomes, indicating that overall results are not affected by genes potentially missing in incomplete assemblies. Thus, including draft genomes allows for a more comprehensive genomic analysis of the genus without affecting its accuracy.

**Fig. 2:**
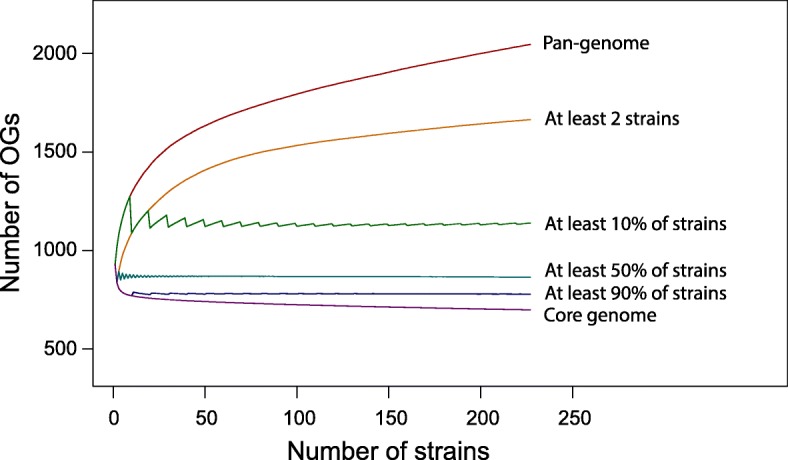
Numbers of Orthologous Groups (OGs) present in a given fraction of chlamydial genomes as dependent on the number of sequentially added strains (saturation curves). The topmost curve shows the pan-genome size, the lowest curve shows the core genome size, and the remaining curves show OGs present in the given fractions of pan-genome (from top to bottom: any 2 genomes, 10%, 50% and 90% of genomes, respectively). Each dot represents a mean value obtained from 500 random permutations of the strain addition order. The jagged pattern is a consequence of the rounding procedure

The total number of genes in the chlamydial pan-genome is expected to increase with addition of new genomes as reflected by the top curve in Fig. 2. The pan-genome of the genus is thus formally open (Additional file 8: Figure S4A), and the Chao lower bound estimate [42] of the pan-genome size is 2909 genes. The pan-genome growth, is, however, completely explained by singletons and the fact that eight species are represented by single genomes in our dataset. In particular, the number of OGs present in at least two strains reaches plateau (second curve from the top in Fig. 2), characteristic of a closed pan-genome (Additional file 8: Figure S4B) with the Chao lower bound estimate of 1672 genes. Overall, the percentile pan-genomes [34] corresponding to OGs present in at least a given fractions of strains, saturate after just a few initial strains (Fig. 2). This shows that we do not expect many changes in the composition of chlamydial core and periphery genomes upon sequencing new strains from the same species.

### Functional annotation shows low diversity of the *Chlamydia* metabolism

Since the chlamydial core genome is conserved across species separated by millions of years of evolution [2], we expect it to contain the most essential gene functions. Indeed, there are apparent differences between fractions of the chlamydial pan-genome (inset table in Fig. 1). Most of core genes encode proteins with conserved domains according to the Pfam database [43], and about half of them are associated with Gene Ontology terms [44]. On the contrary, peripheral genes and singletons often don’t contain conserved domains and functional annotation. In addition, median protein length in the core is 308 amino acids, which is typical for bacterial genes [45]. Non-universal genes are on average shorter, with median lengths of 116 and 64 amino acids for periphery and singletons respectively.

**Fig. 3:**
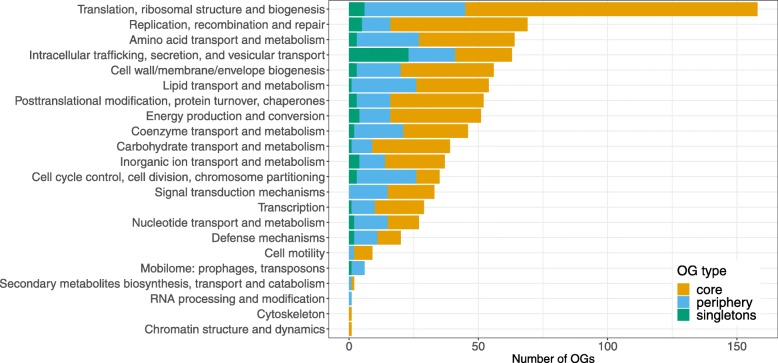
Functional annotation with Clusters of Orthologous Genes (COGs) assigned to OGs from the core, periphery, and singletons. Excluded COGs: Function unknown (S) and General function prediction only (R). In addition, 124, 663, and 313 OGs from the core, periphery, and singletons respectively were not assigned to any COG

Next, we used COGnitor [46] to assign genes to functional categories based on annotated Clusters of Orthologous Genes (COGs). As a result, 517 core, 279 peripheral, and 64 singleton OGs were assigned to 22 categories (Fig. 3 and Additional file 9: Table S5). Based on these annotations, the majority of chlamydial core genes are responsible for the processes related to translation, ribosome biogenesis, replication, recombination and DNA repair (Fig. 3). Contribution of periphery is relatively high for organic molecules’ transport and metabolism. Singletons contribute significantly only to intracellular trafficking, secretion, and vesicular transport.

Based on the gene functional annotations, all studied strains lack pathways to synthesize the majority of amino acids while presumably preserving mechanisms for performing glycine-serine interconversion as implied by the presence of serine hydroxymethyltransferase gene in the core genome. The core genome also includes pyridoxal phosphate-dependent aminotransferase, possibly involved in the amino acid metabolism. The energy metabolism is similar among all *Chlamydia*. All studied strains have a complete glycolysis pathway (with the exception of hexokinase) and the hexose phosphate transporter HPTcp, allowing *Chlamydia* to use glucose-6-phosphate as an energy source. In addition, all strains have ADP/ATP translocase Npt1 providing an alternative pathway to obtain ATP. These results confirm earlier observations in some strains and species of *Chlamydia* [47–50].

Some very common protein families are absent from the chlamydial pan-genome. For example, all *Chlamydia* lack GTP diphosphokinases (Pfam domain families PF04607 and PF13328) and are probably unable to regulate gene expression by (p)ppGpp. Moreover, *Chlamydia* have also lost the omega subunit of RNA polymerase (PF01192) known to participate in binding (p)ppGpp [51]. All *Chlamydia* lack cell division protein FtsZ (PF12327, PF00091) and classical peptidoglycan transglycosylase domains (PF00912), which is not surprising in the light of the unique division mechanism of *Chlamydia* [52, 53]. Another notable loss, which could be explained by stable temperature conditions within host cells, is cold shock proteins (PF00313) that weakly bind to single stranded RNA and destabilize RNA secondary structures [54, 55].

Non-universal genes were mapped to several metabolic pathways, including biosynthesis of biotin, tryptophan, thiamine, folate, purines, and pyrimidines. The non-universal distribution of some enzymes from these pathways has been shown to play a role in tissue tropism and persistent infections [2, 56, 57]. In particular, *C. trachomatis*, *C. suis*, *C. pecorum*, and *C. muridarum* strains lack phenylalanine hydroxylase responsible for the biosynthesis of tyrosine from phenylalanine. *C. pecorum*, *C. felis* and *C. caviae* have a complete pathway of tryptophan production from kynurenine, while *C. suis* and genital strains of *C. trachomatis* are able to produce tryptophan from exogenous indole, *cf.* [32].

### Strong purifying selection in the core and signatures of ongoing genome streamlining

**Fig. 4:**
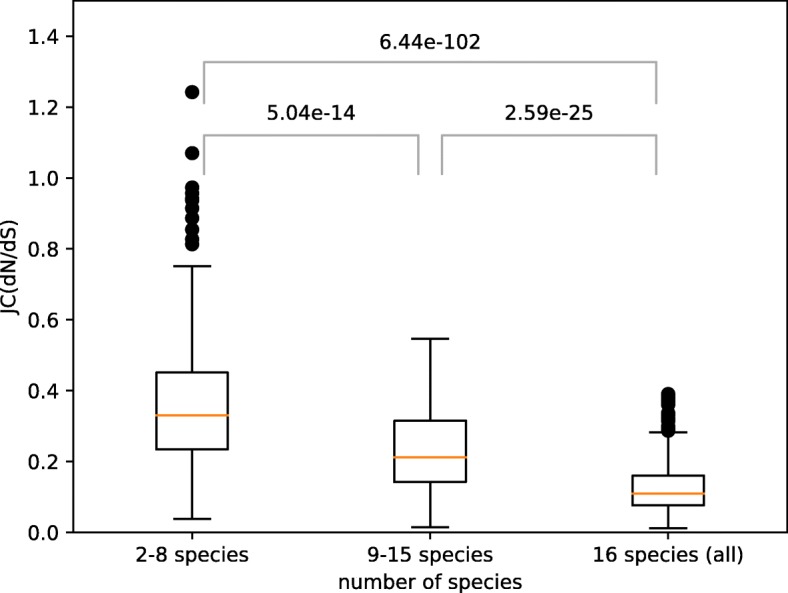
Nonsynonymous to synonymous substitutions ratio (dN/dS) as dependent on the number of species that have representatives of an Orthologous Group (OG). Multiple substitutions per site are allowed (the Jukes-Cantor distance correction). The difference between medians is estimated with the nonparametric Mann-Whitney U test with the Bonferroni correction for multiple testing

To test whether the chlamydial core and periphery exhibit different patterns of selection, we estimated nonsynonymous to synonymous substitution ratio between species (dN/dS). For the dN and dS calculations, we performed 30 rounds of random selection of pairs of strains from two different species. The median dN/dS ratio was then assigned to the respective OGs. Our results showed that the purifying selection was stronger for genes present in a higher number of species, with dN/dS ratio being the lowest in the core (Fig. 4). This finding further supports the distinction between the highly conserved core and the evolvable periphery.

To find the signatures of ongoing genome streamlining, we next analyzed the presence and distribution of frameshift and nonsense mutations in the chlamydial pan-genome. These two types of mutations were predicted using the RAST “fix frameshift” option as a part of the de novo genome annotation pipeline. To discriminate mutations from sequencing errors, we only considered events present in at least two genomes from the same OG. In addition, in this section we only consider frameshift and nonsense mutations located more than 60 nucleotides away from gene end (as potentially more harmful) and refer to the genes harboring these mutations as putative pseudogenes. The complete list of predicted frameshift and nonsense mutations with their location relative to the gene start and end is provided in Additional file 10: Table S6.

The estimated number of pseudogenes in each individual genome is rather low, ranging from 9–12 in *C. trachomatis*, *C. muridarum*, and *C. suis* to 21 in *C. pneumoniae* and 28–29 in *C. abortus* and *C. psittaci* (Additional file 5: Table S4). However, in total, predicted pseudogenes are present in 297 OGs, including 176 core orthologous group. None of these OGs were pseudogenized in the whole genus, while at least 26 cases can be considered species-specific (21 more OGs contained putative pseudogenes in all but one genome of some species, Additional file 11: Table S7). Other frameshift and nonsense mutations were present in the subsets of strains, suggesting that the majority of detected events happened after speciation.

OGs with high number of pseudogenes often contained hypothetical proteins. Some genes can be, however, mapped to several biological pathways, such as the TCA (tricarboxylic acid) cycle, homologous recombination, amino acid and nucleotide metabolism, indicating potential hotspots of further genome reduction in some species. One described case is parallel elimination of arginine: agmatine exchange system genes (*aaxABC* gene cluster) in *Chlamydia trachomatis* [58]. In our dataset, all strains of *Chlamydia trachomatis* serovars L1-L3 had nonsense mutation in the arginine decarboxylase gene (OG457, *aaxB*), and 13 strains from other serovars had frameshifts in the arginine/agmatine antiporter (OG458). In addition, all *Chlamydia abortus* strains had missense mutation in *argR* encoding the arginine pathway regulatory protein (OG982). Of note, this gene is completely absent in the genomes of *C. trachomatis*, *C. muridarum*, *C. suis*, *C. avium*, and *C. gallinacea*. Taken together, these results suggest elimination of arginine-related metabolic processes in multiple Chlamydial species.

Another example is apparent parallel elimination of genes involved in TCA cycle across *C. trachomatis*. Core gene coding for fumarate hydratase, class II (OG134) harbors nonsense mutation in 8 trachoma strains (serovars A-C) and frameshift in 22 LGV strains (L1-L3) and one urogenital strain (D-K). Succinate dehydrogenase flavoprotein (OG671, core) contained nonsense mutation and frameshift in 8 and 46 different strains of *C. trachomatis*, respectively. Succinate dehydrogenase iron-sulfur protein (OG670, core) contained nonsense mutation in all 110 *C. trachomatis* genomes. Succinate dehydrogenase cytochrome b558 subunit (OG672, core) was pseudogenized in 108 out of 110 *C. trachomatis* genomes (both frameshift and nonsense mutations in most strains). In addition, the succinate dehydrogenase flavoprotein gene contained nonsense mutation in one genome of *C. suis* (strain 30-22b) and both frameshift and nonsense mutations in one genome of *C. psittaci* (strain Po_An), and the gene of succinate dehydrogenase iron-sulfur protein harbored frameshift in one out of three genomes of *C. muridarum*. Other genes from the TCA cycle are missing in the genus, including aconitate hydratase, isocitrate dehydrogenase, and 2-oxoglutarate dehydrogenase E1 component. This again might indicate reduced purifying selection on genes involved in the TCA cycle in multiple species. Of note, none of the core OGs discussed above show an increased dN/dS ratio compared to other core genes (Additional file 12: Figure S5), which is consistent with results in [58] for arginine: agmatine exchange system genes and might be explained by recent inactivation of these genes.

Finally, large numbers of putative pseudogenes were characteristic of several virulence-related OGs containing many paralogous genes per genome or belonging to expanded protein families (see below). Notable examples are genes encoding polymorphic membrane protein (OG1; 508 genes in 82 genomes; 56 genomes with putative pseudogenes), cytotoxin (OG3; 452 genes in 181 genomes, 40 genomes with putative pseudogenes) and inclusion membrane protein (OG744; single copy in 225 genomes, 58 genomes with putative pseudogenes). Complete information about predicted frameshift and missense mutations is provided in Additional file 10: Table S6 and Additional file 11: Table S7.

### Low contribution of gene loss and genomic rearrangements to chlamydial phenotypic diversity

**Fig. 5:**
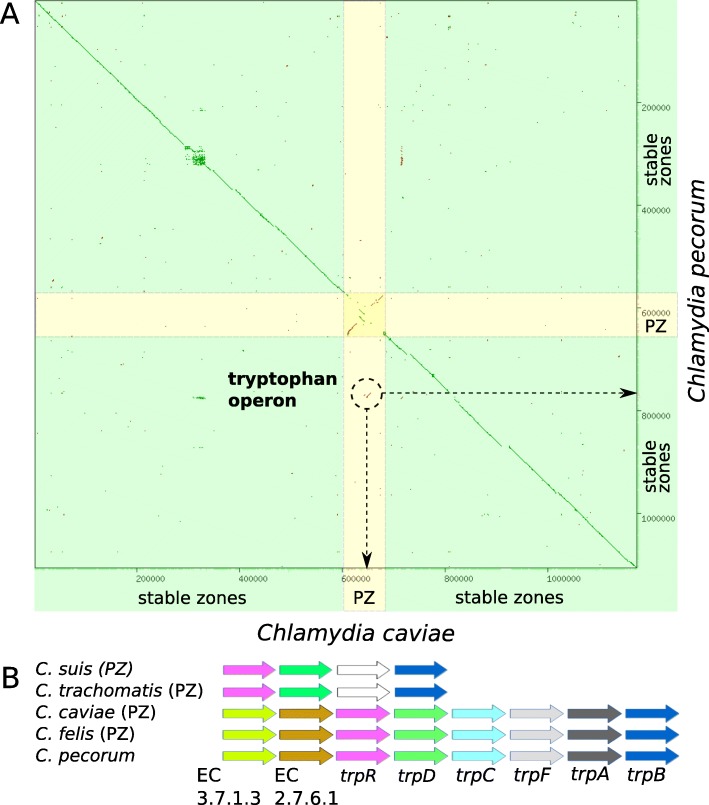
Structure of the tryptophan operon in *Chlamydia* spp. **a** Graphical full-genome comparison of *C. caviae* GPIC and *C. perocum* E58, the dots coordinates correspond to the positions of the homologous fragments in the genomes (bp scale). Location of the dots on the diagonal line shows the similarity of gene order for most genes. The exceptions are the genes involved in rearrangements in the Plasticity Zone (the segment labeled PZ and marked by yellow color) and the fragment containing the tryptophan operon (marked by dashed-line circle and arrows). **b** Genes of the tryptophan operon found in different *Chlamydia* spp., the rows order shows the order of genes in the chromosomes. The same genes have the same color, white color corresponds to genes with unknown functions

The reconstruction of genome rearrangements in analyzed species shows that the gene order in the *Chlamydia* chromosomes is highly conserved as only species-specific large-scale inversions and translocations were detected (Additional file 13: Figure S6a). The larger set of inversions (52 events) differentiates the *C. suis/C. trachomatis/C. muridarium* group from other species, while within these species the genomes are strongly collinear. Thirteen inversions were found on other branches, however these rearrangements are shorter and mainly occur in the plasticity zone (Fig. 5a). Topology of the phylogenetic species tree constructed based on gene order only slightly differs from the one of the tree constructed based on gene sequences alignment indicating high level of similarity of gene order in closely related species (Additional file 13: Figure S6b, Additional file 14: Figure S7). The gene order outside the plasticity zone in the strains of the same species is stable even for rare periphery genes.

Highly optimized chlamydial pan-genome is characterized by a very low number of species-specific losses indicating that a major genome reduction has been completed prior to speciation (0–5 uniquely absent genes per species, Additional file 5: Table S4). Yet, there are multiple cases of genes absent in some groups of strains. Out of 341 analysed peripheral OGs, 131 groups had a mosaic pattern (polyphyletic distribution), meaning that the presence of such a gene cannot be explained by a single gene gain or loss event. For example, phosphoglycolate phosphatase (OG799) is missing in 16 genomes of *C. psittaci*, 9 genomes of *C. suis*, and 2 genomes of *C. gallinacea* (considering only complete genomes). Genes encoding ribosomal protein L35p (OG796) are missing in 31 genomes of *C. trachomatis* and 4 genomes of *C. psittaci*, and genes of ribosomal protein L33p (OG875) are missing in 88 genomes of *C. trachomatis* and 10 genomes of *C. pneumoniae*. The distribution of all orthologous groups with mosaic phyletic patterns across species is provided in Additional file 15: Table S8 (only complete genomes and species with at least 2 complete genomes) and Additional file 16: Table S9 (all genomes).

To further describe the evolution of genes with mosaic phyletic patterns in the *Chlamydia* genomes, we used the following evolutionary model combining information about the gene’s genomic location and phylogenetic tree. If a gene with a mosaic phyletic pattern has been inherited vertically from the common ancestor and lost by several genomes, we expect to find it at the same syntenic region in the remaining strains. Genes not satisfying this condition are candidates for having been obtained horizontally. For this analysis, we excluded genes whose universal neighbors were affected by the reconstructed rearrangements, that is, genes located at or near boundaries of synteny blocks. In addition, the phylogenetic gene trees for the analyzed orthologous groups were checked to see whether each species were monophyletic, that is, formed a separate branch in these trees (Additional file 17: Table S10).

For most peripheral genes with mosaic phyletic patterns, phylogenetic gene trees were consistent with the species tree. Combined with the conserved genomic location of genes from these OGs, this allowed us to exclude inter-species horizontal gene transfer and suggested independent losses as the most likely evolutionary scenario. Most of cases of trees inconsistency were explained by short tree branches due to low divergence of gene sequences in close *Chlamydia* species such as *C. trachomatis / C. muridarium*. Hence they cannot be interpreted with confidence.

One interesting case of not-trivial gene history, involving likely horizontal transfers, is a DNA fragment containing eight genes related to the tryptophan biosynthesis and metabolism. These genes form a stable genome locus in *C. pecorum*, whereas in *C. caviae* and *C. felis* the fragment is located in the plasticity zone (Fig. 5a). Detection of several genes from the fragment mixed up with some species-specific genes in the plasticity zone in *C. trachomatis* and *C. suis* allows us to suggest horizontal transfer in the evolutionary history of the fragment (Fig. 5b) even though the phylogenetic analysis does not reveal the source of the transfer.

Overall, the majority of polyphyletic OGs cannot be explained by the lateral gene transfer. Their distribution is hence most parsimoniously explained by parallel gene losses due to ongoing genome streamlining. Another possibility would be divergence of some groups of orthologous genes into separate orthologous groups due to increased evolutionary rate at some branches (some examples are discussed below).

### The chlamydial genomic diversity is mainly contained within several gene families and results from paralogisation

**Fig. 6:**
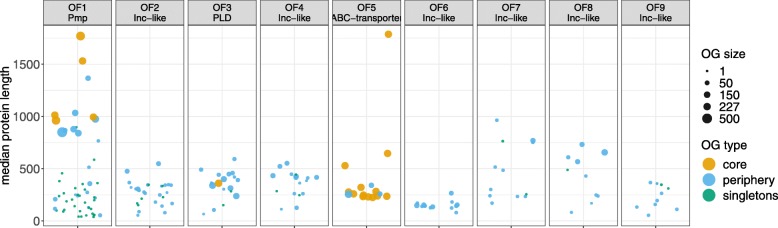
Orthologous families (OFs) with ten or more member OGs. Each dot corresponds to an individual OG, the dot size reflecting the number of genes in OG (continuous scale). The Y-axis stands for the median length of the encoded proteins (in amino acids). Color represents the OG type (core, periphery, or singletons). The complete list of OFs is provided in Additional file 18: Table S11

To further explore the evolutionary history and relationships among orthologous groups, we performed MCL clustering (implemented in the orthoMCL pipeline) using one representative protein from each OG. As a result, 666 OGs were clustered into 198 orthologous families (OFs), containing 2 to 54 OGs each (Additional file 18: Table S11). The largest OFs are annotated as polymorphic membrane proteins, *phospholipase* D, ABC transporters, and multiple groups of inclusion membrane proteins (Fig. 6). Most OGs within families vary dramatically in terms of protein lengths and distribution across the genus, reflecting the processes of paralogisation, functional divergence, as well as pseudogenization.

The largest OF (OF1 with 54 OGs) was formed by the superfamily of polymorphic membrane proteins (Pmps), identified by the presence of characteristic protein domains: PF03797 (Autotransporter), PF07548 (*Chlamydia* polymorphic membrane protein middle domain), and PF02415 (*Chlamydia* polymorphic membrane protein repeat) [59–62]. Pmps are surface-exposed proteins involved in host-cell interactions and virulence [29, 60, 63–65]. This expanded superfamily was shown to be vertically inherited and conserved among *Chlamydiae*, *Verrucomicrobia*, *Lentisphaerae*, and *Planctomycetes* [66]. In agreement with that, all Pmps have clustered in a single OF in our analysis. This family is particularly expanded in some species, with the number of Pmps ranging from 8–11 in *C. trachomatis*, *C. muridarum*, and *C. suis* to 16–25 in *C. abortus*, *C. psittaci*, and *C. pneumoniae* (Additional file 5: Table S4). Overall, OF1 contains 5 core, 18 peripheral, and 31 singleton OGs featuring a with wide range of gene lengths and OG sizes, and with rare OGs being on average much shorter (Fig. 6, the first panel). Of note, not all members of this OF are functional Pmps, though all seem to originate from the same gene superfamily. Many genes with the encoded protein lengths below 600 amino acids lacked some or all characteristic Pfam domains, and potentially represent pseudogenes or genes that gained new functions after duplication events.

Multiple OFs contained groups of inclusion membrane proteins (Incs). These are virulence-related genes unique to phylum *Chlamydiae* [67]. Incs do not form a single gene family and are distinguished by their localization within the inclusion membrane rather than by sequence similarity [30, 67, 68]. Here, we identified 332 OGs containing genes with sequence similarity to previously annotated Incs from six chlamydial species [25]. Since precise identification of Incs requires analysis of protein secondary structures, which was beyond the scope of this study, we refer to these groups as Inc-like OGs. 216 of Inc-like OGs were clustered into 54 OFs, containing 2 to 29 OGs each (Fig. 6 and Additional file 18: Table S11). The number of Incs-like genes per genome varies significantly by species: from 65–70 in *C. trachomatis*, *C. suis*, and *C. muridarum* to 102–110 in *C. psittaci* and *C. abortus* and up to 140–147 in *C. pneumoniae* (Additional file 5: Table S4), accounting 6% to 14% of the total coding capacity in the respective species.

The third largest OF (OF3, 19 OGs) was annotated as *phospholipase D* (PLD) based on the presence of a characteristic PLD-like domain PF13091 [69]. PLD also is an expanded gene family in *Chlamydia* linked to virulence [31, 57, 70]. This OF contains one core gene (OG221) as well as multiple paralogs in the plasticity zone of *C. trachomatis*, *C. muridarum*, *C. suis*, and *C. pecorum* (Additional file 5: Table S4). The plasticity zone complement of *phospholipase* D genes has been shown to represent an important strain-specific virulence factor [31]. Of note, the chlamydial pan-genome contains one more core OG with a PLD-like domain (OG377, assumed to encode cardiolipin synthase), which is present as a single copy per genome and not clustered with other OGs.

Several other gene families clustered into OFs include ATP-binding proteins of ABC transporters (OF5), aminoacyl-tRNA synthetases (OF29, OF43, OF46), and translation elongation factors (OF30). More examples can be found in Additional file 18: Table S11.

Four OFs contained OGs which represented orthologs divergent between early branching chlamydial clades corresponding to *Chlamydia* and *Chlamydophila* in division proposed by [40]. One interesting case is chlamydial GroEL protein. All *Chlamydia* are known to possess three paralogs of the *groEL* gene that diverged functionally after gene duplication events [71]. In our dataset, two *groEL* paralogs belong to the core genome (OG45 and OG245), while the third was split into two OGs between the two clades (OG847 present in all *C. trachomatis*, *C. muridarum*, and *C. suis* and OG954 present in all other species). This indicates strong sequence divergence of one *groEL* gene between clades resulting in one orthologous group being split into two OGs by the orthoMCL algorithm. These 4 OGs were clustered in a single orthologous family (OF32). A similar split happened in three putative inclusion membrane proteins (OF148, OF36, and OF121).

Finally, several OFs contained core, or widespread peripheral, genes together with their truncated paralogs, which were present in a few strains. For example, OF62 contains the core gene encoding succinate dehydrogenase cytochrome b558 subunit (286 amino acids, OG672), one shortened paralog presents in seven genomes (189 amino acids, OG1848), and another one present in two genomes (39 amino acids, OG2339). Another example is OF116 containing core gene coding for Type IV pilus biogenesis protein PilO (905 amino acids, OG535) and its truncated paralog present in 3 genomes (196 amino acids, OG2159).

The origin of multiple non-universally conserved OGs can be therefore traced back to core genes and several expanded paralogous gene families suggesting limited pool of new genes in chlamydial pan-genome.

### A subset of the pmpG group of polymorphic membrane proteins demonstrates a combination of phase variation, gene duplication, and pseudogenisation processes

The largest OG in our analysis (OG1, 508 proteins) contained a subset of the PmpG group of polymorphic membrane proteins [72] in twelve chlamydial species (all except *C. trachomatis*, *C. muridarum*, *C. suis*, and *C. avium*). For simplicity, we annotated this OG as PmpG, keeping in mind that it also contains other Pmps highly similar in sequence to annotated PmpGs. The entire family of Pmp proteins is related to virulence, and the PmpG sub-group has been shown to act as adhesins in vitro [59]. The subset of *PmpGs* clustered into OG1 is of particular interest because it contains known cases of frameshifts due to the length variation of polyG tracts in *C. pneumoniae* [61] and *C. abortus* [60]. The replication-coupled frameshifting in homopolymeric tracts (slipped strand mispairing) is a mechanism of phase variation, high-frequency on/off switching of phenotype expression involved in the host adaptation and immune evasion [73].

Our analysis shows extensive paralogisation and pseudogenisation within OG1, reflecting reduced evolutionary constraints on this group of genes. The number of paralogs per genome ranges from two to twelve with varying diversity within species (3–8 genes per strain in *C. psittaci* versus 10–12 genes per strain in *C. pneumoniae*. In total, 82 out of 508 encoded proteins lack a predicted autotransporter domain. Out of the former, 56 genes encode truncated proteins (308–591 amino acids long versus median length of 849 for this OG). Among the 26 full length proteins lacking autotransporter, 20 belong to *C. pecorum* probably indicating sequence divergence within this group. Out of the remaining 426 proteins with a predicted autotransporter domain, 23 with length between 273 and 342 amino acids lack both middle and repeat Pmp domains and 27 more proteins of 383–636 aa lack only N-terminal repeat domains (Additional file 19: Table S12). They are presumably pseudogenes encoding truncated proteins.

Out of the remaining set of 374 full-length genes, 121 genes have frameshift or nonsense mutations, with the number of events ranging from 1 to 9 per protein. Interestingly, in 109 genes, frameshifts happened within the distance of 5 nucleotides from the homopolymeric tracts (Additional file 20: Table S13) suggesting the slipped strand mispairing mechanism. In particular, the majority of events have happened in the vicinity of long polyG tract of varying length in *C. psittaci*, *C. abortus*, and *C. felis*: 86 frameshifts in the interval of 1296-1326 nucleotides from gene end; polyG tracts of 8–19 bp in the same region). Other frameshift hotspots in *C. pneumoniae* genes are polyC of length 13–24 (7 frameshifts, 507–516 nucleotides upstream of the gene end), polyG of length 13 (2 frameshifts, 1866 nucleotides upstream of the gene end), and polyA of length 5 (11 frameshifts, 2304 nucleotides upstream of the gene end).

**Fig. 7:**
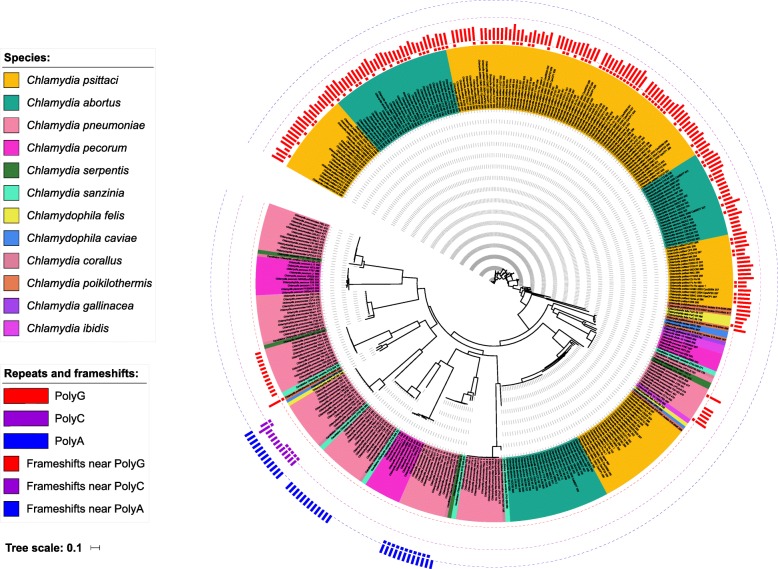
Phylogenetic tree for OG1 (a subset of the PmpG group of Polymorphic membrane proteins). The tree was constructed based on amino acid sequence of the autotransporter domain (PF03797.15) of 374 full-length proteins from OG1. The leaves are colored by species. Homopolymeric tracts (polyA, polyG, and polyC) are shown as colored bars near the leaves with length proportional to the tract length. Dots under the bars represent frameshifts within 5 nucleotides from the tract

We visualized the relationship between the presence of homopolymeric tracts and frameshifts on the phylogenetic tree (Fig. 7) constructed based on the amino acid sequence of the autotransporter domain, which is the slowest evolving part of Pmps [63, 72]. Since all frameshifts at polyN tracts are located upstream of the autotransporter domain, one would expect true pseudogenes to accumulate mutations faster and hence be located on longer branches. Furthermore, if frameshifts are rare events, one would expect sequences with the same polyN length to cluster together.

In the phylogenetic tree (Fig. 7), we can see genes from the same species forming multiple clusters likely indicating distinct paralogs within the orthologous group. In the lower part of the tree, several clusters have homopolymeric tracts corresponding to the frameshift hotspots in *C. pneumoniae*. In both cases of polyG and polyC tracts, genes with and without frameshifts cluster together and have homopolymeric tracts of variable length, suggestive of multiple events due to the slipped strand mispairing. In contrast, the polyA tract has fixed length in all genes, and frameshifts in 11 genes likely represent a single event in the common ancestor.

Paralogs of *PmpG* in *C. psittaci*, *C. abortus* and *C. felis*, clustering with *C. pneumoniae* genes do not contain homopolymeric tracts. However, in the upper part of the tree, we can see further expansion of the *pmpG* genes in these species, which was likely recent as indicated by very short branches compared to the bottom part. Most of these genes have polyG tract approximately 1300 nucleotides upstream of the gene end (which is absent at that position in *C. pneumoniae*), and more than half of these genes contain frameshifts near polyG. The length of the polyG tract is highly variable, and genes do not cluster either by the presence of frameshift or by the length of the homopolymeric tract. This is consistent with the model of frequent on/off switching due to phase variation. If this is the case, then frameshifted genes are not fully pseudogenized but rather temporary switched off, and therefore do not accumulate a higher number of mutations in the autotransporter domain.

PmpGs of *C. trachomatis* cluster in a distinct OG present as a single copy throughout the genus (OG25). These genes also contain a polyG tract of varying length close to the gene start, though all are in frame (except for a single frameshift in *C. psittaci* VS225 outside the homopolymeric tract). In in vitro setups, the percentage of *C. trachomatis* inclusions not expressing PmpG varied from 1% to 10% in several independent experiments performed under the same infection conditions [65], hence suggesting a phase variation mechanism with frameshifting happening in a minor fraction of the clone populations and thus not detected in the sequenced genomes. In contrast, OG1 was found to be the only Pmp group in *Chlamydia* featuring multiple independent frameshifts along with high variation of polyG/C lengths. This might result from a reduced selection pressure on this OG due to extensive paralogisation. Thus, if frameshifted genes can spontaneously revert back to the functional state when the polyN length changes, different combination of Pmps from this group will be exposed on the cell surface within and between the clonal populations. Since the *pmp* genes are known to exhibit high sequence variation in their N-terminal part (the region with Pmp repeats) [65], this would allow for higher phenotypic diversity beneficial for the immune evasion.

## Discussion

Comparative genomic analysis of 16 currently recognized or candidate species from genus *Chlamydia* shows that their pan-genome is characterized by a large pool of universally conserved core genes, a small periphery, and a few unique strain-specific genes. Our results are consistent with earlier studies with much smaller sample sizes [21, 37]. Moreover, since the pan-genome of *Chlamydia* is closed after accounting for singletons, we do not expect many changes in the composition of chlamydial core and periphery upon sequencing new strains. Overall, the pool of chlamydial genes is limited due to their isolated intracellular lifestyle. Our analysis suggests that the majority of gene gains are due to paralogisation and sequence divergence of some commonly present genes and paralogous gene families, including Pmps, PLDs, and Incs.

The structure of the pan-genome reflects differences in evolutionary constraints among chlamydial genes. The core is mainly comprised of genes responsible for the information-processing machinery and central metabolism and evolves under strong purifying selection. The periphery, on the other hand, contains many virulence-related genes and evolves under reduced purifying selection. In addition to the highly conserved set of core genes, the chlamydial genome is characterized by remarkable collinearity and low number of genomic rearrangements. Generally, a high rate of rearrangements such as inversions and large deletions is usually associated with accumulation of mobile elements accompanying adaptation to a new environment. A notable example is adaptation to intracellular lifestyle of *Burkholderia mallei* that descends from soil-dwelling pathogen *Burkholderia pseudomallei* [74]. This phenomenon is traditionally explained by weaker selection against repetitive elements due to the decreased effective population size [75]. The chlamydial genomes have few mobile elements and demonstrate a high level of conservation of the gene order in strains of the same species indicating that the genome reduction by deletions may have happened during the adaptation to the intracellular lifestyle but had been completed prior to speciation.

The estimated number of frameshift and nonsense mutations in each individual genome is rather low, which agrees with earlier studies [2, 19]. However, in total, putative pseudogenes are present in multiple OGs, and are particularly abundant in several biological pathways and gene families. Yet more pseudogenes have been detected as short reading frames homologous to other full length genes after clustering genes into orthologous groups and orthologous families. Furthermore, we have observed multiple cases of parallel gene losses happening after the speciation. This agrees with the model of genome streamlining where non-functional genes are expected to be removed from the genomes under strong purifying selection [16]. In addition, large numbers of putative pseudogenes are characteristic of several virulence-related OGs, which might be a side effect of their frequent paralogisation, as well as antigen and phase variation under strong positive selection from the host immunity [28–30, 64, 68].

## Conclusions

Integrating information from more than two hundred genomes from sixteen chlamydial species provides us with an increased power to detect ongoing processes of genome streamlining, on one hand, and expansion within several paralogous gene families, on the other, both representing relatively rare events in each individual genome. This supports the notion of the dynamic stability of prokaryotic genomes and reflects unique properties of chlamydial genomes shaped by the selective pressure towards genome reduction due to intracellular lifestyle and by evolutionary success in host niche adaptation and immune evasion.

## Methods

### Data selection and pre-processing

A total of 262 genomes of genus *Chlamydia* were downloaded from NCBI Genbank [76]. The sample included complete genomes, as well as draft genomes assembled into at most 10 contigs, as of April 2019. We excluded genomes sequenced after in vitro mutation or recombination experiments (e.g. [77]) and genomes with reported assembly anomalies [78]. If two genomes were assigned to the same strain or were identical based on the concatenated sequence of all universally conserved genes within the genus, only one genome from a pair was retained. The final sample was comprised of 161 complete and 66 draft genomes of genus *Chlamydia*. *Waddlia chondrophila* WSU 86-1044 (NC 014225) was selected as an outgroup to root the phylogenetic tree of the genus. The analyzed genomes are listed in Additional file 1: Table S1.

The analysis of chlamydial plasmids was beyond the scope of this study. However, some draft genome assemblies contained plasmid sequences as separate contigs. To exclude them, we performed BLASTn [79] (the megablast algorithm) on genomes using a set of annotated chlamydial plasmids in complete genomes from the sample with the e-value threshold 10^−3^. All contigs that had sequence identity of at least 98% and coverage along the contig length of at least 98% with at least one plasmid from the dataset were discarded.

### Genome annotation

To exclude biases arising from differences in gene prediction algorithms, genes in all selected genomes were re-annotated de novo using the RAST pipeline with the “fix frameshifts” gene caller option [39]. Gene functions were assigned by RAST. All predicted CDSs were scanned for the presence of conserved protein domains (Pfam-A database [43], release 30.08.2018) using HMMER 3.1b1 [80]. The assignment of Enzyme Commission (EC) numbers and Gene Onthology (GO) terms to individual genes was included in the RAST annotation pipeline.

### Building the pan-genome: orthologous groups and orthologous families of genes

Orthologous groups (OGs) of genes were identified using the orthoMCL v.2.0.9 software package (default parameters, e-value cutoff of 10^−5^, percent match cutoff 50%, mcl algorithm inflation value 1.5) [38]. More distant relationships among OGs were determined by an additional round of MCL clustering based on all-vs-all BLASTp results (using the same cutoff parameters and inflation value as the ones implemented in OrthoMCL) applied to one representative from each OG (the one with the length closest to the group median) which resulted in clustering OGs into so called orthologous families (OFs).

A function was assigned to each OG based on the most frequent predicted function of its members in the RAST annotation. To remove potential false positives of the gene calling algorithm, OGs containing only hypothetical proteins with median length below 50 amino acids were excluded from subsequent analysis. OGs were assigned to Clusters of Orthologous Genes (COG) using the COGnitor software [46]. Putative inclusion membrane proteins (Incs) were predicted based on sequence similarity to previously identified Incs [30]. Amino acid sequences for the list of 461 Incs annotated in six chlamydial species (*C. trachomatis*, *C. pneumoniae*, *C. felis*, *C.abortus*, *C. caviae*, and *C. muridarum*) were downloaded from NCBI Genbank and scanned against representatives of OGs using BLASTp (e-value cutoff 0.01, coverage cutoff 50 amino acids). Putative polymorphic outer membrane proteins (Pmps) were predicted based on the presence of characteristic Pfam domains PF03797.15 (Autotransporter domain), PF07548.7 (*Chlamydia* polymorphic membrane protein middle domain), and PF02415.13 (*Chlamydia* polymorphic membrane protein repeat). Putative *phospholipase* D endonuclease superfamily (PLD) proteins were annotated based on the presence of PLD-like domain PF13091.6. The resulting lists of annotated OGs and OFs are provided in Additional file 2: Table S2 and Additional file 18: Table S11 respectively.

### Prediction of frameshift and nonsense mutations

Frameshifts and internal stop codons (nonsense mutations) were predicted within the RAST annotation pipeline by enabling the “fix frameshifts” option, and the coordinates of frameshifts were defined relative to the gene start and end. In the original algorithm, this option is mainly targeted at detecting frameshifts caused by sequencing errors [81]. However, since it is highly unlikely that an error in the same CDS has occurred in two independent sequencing experiments, we considered events present in at least two genomes in the same OG as true pseudogenisation events. With this approach, we have limited power to detect rare events in species represented by few genomes, but this is essential to avoid false positives. The only exception was made for the analysis of frameshifts near polyG tracts. Since these sequences are known to be mutation-prone [82], and pmp genes truncated at polyG tracts have been detected in *Chlamydia* in in vivo experiments [60, 61], there events were considered separately.

The distribution of these events was found to be nearly uniform along the gene length (p=0.5906, Kolmogorov-Smirnov test), therefore we could not set an obvious cut-off for non-deleterious events. For the purpose of functional analysis, we only focused on frameshift and nonsense mutations located at least 60 nucleotides upstream of the gene end (as potentially more deleterious). For reconstruction of the chlamydial phylogenetic tree, all OGs with predicted frameshift and nonsense mutations were excluded. All predicted frameshift and nonsense mutations are listed in Additional file 10: Table S6.

### Alignments and phylogenetic analysis

Nucleotide alignments were constructed using the MACSE [83] algorithm. The phylogeny was reconstructed using the Fasttree software (GTR+CAT model) [84]. For an unrooted phylogenetic tree, we used the concatenated nucleotide sequence of the genes that were shared by all 227 strains, were present as one copy per genome, and contained no predicted frameshifts or nonsense mutations. For a rooted tree (Additional file 14: Figure S7), we added a requirement for universal genes to be also shared (single copy per genome, no predicted frameshifts or nonsense mutations) with the outgroup, *Waddlia chondrophila* WSU 86-1044; thus only 302 genes remained. Phylogenetic trees were visualized using the iTOL online tool.

For comparing distribution of individual OGs with phylogenetic tree of the genus (Additional file 14: Figure S7), we excluded draft genomes and species with less than two complete genomes, and only considered OGs present in at least two species and absent in two or more genomes (341 OGs in total). Analysis was done using ete2 python package [85].

### Reconstruction of genomic rearrangements

Synteny blocks were constructed using the ProgressiveMauve software [86] with standard parameters. Stable common gene blocks, i.e. groups of common genes that maintain the same order across all considered genomes, were reconstructed using the DRIMM-Synteny algorithm [87] for single-copy genes universally conserved in these genomes. The phylogenetic tree based on gene order was constructed by MLGO algorithm [88]. The evolutionary history of inversions was reconstructed by MGRA 2.2 [89].

### dN/dS calculations

To estimate the nonsynonymous to synonymous substitution ratio between (dN/dS) within (pN/pS) species, we used the KaKs Calculator Toolbox version 2.0 with default parameters [90]. Multiple substitutions were accounted for by applying the Jukes-Cantor correction [91]. Only orthologous groups containing no paralogs were considered. For the dN and dS calculations, we performed 30 rounds of random selection of pairs of strains from two given species. The median dN/dS ratio was then assigned to respective OGs.

### Homopolymeric tracts in OG1

All nucleotide sequences of OG1 were extracted before automatic correction for frameshifts implemented in the RAST pipeline. Sequences were scanned for the presence of homopolymeric tracts comprised of 5 or more nucleotides with no mismatches (Additional file 21: Table S14). Frameshifts located less than 5 nucleotides from homopolymeric tracts were identified and referred to as frameshift hotspots (Additional file 20: Table S13). Since the location of frameshift hotspots was highly conserved, especially relative to the gene end, we next scanned for the presence of corresponding *polyN* tracts in all OG1 genes of the same species in 20 nt regions around the frameshift (coordinates calculated relative to the gene end).

The autotransporter domain, being the most conserved part of OG1, was used to construct the phylogenetic tree. Autotransporter domain sequences were aligned with the Muscle software [92]. The phylogenetic tree for the Autotransporter domain was constructed by the FastTree approximately maximum likelihood approach (JTT+CAT model) [84]. Phylogenetic trees were visualized using the iTOL online tool [93].

## Additional files

Additional file 1 Summary of the analysed genomes. (CSV 24 kb)

Additional file 2 Summary of orthologous groups (OGs) for 227 genomes of genus *Chlamydia*. (CSV 362 kb)

Additional file 3 OGs by their presence in individual genomes. (CSV 926 kb)

Additional file 4 Histogram of 1288 orthologous groups containing only hypothetical proteins with the average length below 50 amino acids by the number of genomes that have these OGs. These OGs were removed as potential false positives of gene prediction algorithms. (PDF 5 kb)

Additional file 5 Pan-genome statistics by species. (CSV 3 kb)

Additional file 6 Distribution of orthologous groups by the number of strains that have them for seven *Chlamydia* species with more than two available genomes. (PDF 8 kb)

Additional file 7 Distribution of orthologous groups by the number of strains that have them based on 161 complete genomes (red) or all 227 genomes assembled in at most 10 contigs (black). The pan-genome structure is not affected by genes potentially missing from 66 draft genomes. (PDF 7 kb)

Additional file 8 The number of new genes added to the pan-genome upon addition of new strains of *Chlamydia* spp. The number of new genes is plotted as a function of the number (*n*) of strains sequentially added (see the model in [94]). For each *n*, points are the values obtained for different strain combinations; red symbols are the averages of these values. The superimposed line is the best fit with a decaying power law *y*=*A*·*n*^*ξ*^. The pan-genome is considered open for *ξ*>−1 and converges to a constant for *ξ*<−1. (A) Full pan-genome, *N*(*n*)=173·*n*^−0.92^, (B) only OGs present in at least two genomes, *N*(*n*)=193·*n*^−1.05^. (CSV 7334 kb)

Additional file 9 Clusters of Orthologous Genes (COGs) and corresponding functional categories assigned to OGs. (CSV 117 kb)

Additional file 10 Predicted frameshift and nonsense mutations in Chlamydial pan-genome. For the analysis of putative pseudogenes, events located less than 60 bp. away from gene end or present in a single genome from the corresponding OG were excluded. (CSV 600 kb)

Additional file 11 OGs with putative pseudogenes by the number of affected genomes in different chlamydial species. Frameshift and nonsense mutations located less than 60 bp upstreamof the gene end or present in a single genome from the corresponding OG were excluded. (CSV 31 kb)

Additional file 12 Nonsynonymous to synonymous substitutions ratio (dN/dS) for the selected core OGs with a high number of frameshift and nonsense mutations (vertical red lines) compared to the overall dN/dS distribution for core genes (histogram). OG134: fumarate hydratase, class II, OG671: succinate dehydrogenase flavoprotein, OG670: succinate dehydrogenase iron-sulfur protein, OG458: arginine/ornithine antiporter, OG457: arginine decarboxylase, OG672: succinate dehydrogenase cytochrome b558 subunit. (PDF 47 kb)

Additional file 13 Rearrangements in *Chlamydia* genomes. a)Full-genome alignment of *Chlamydia* genomes. Reference strains used for synteny blocks construction are *C. trachomatis* D/UW-3/CX, *C. suis* SWA-2, *C. abortus* S26/3, *C. psittaci* 6BC, *C. pneumoniae* CWL029, *C. pecorum* E58, *C. muridarum* Nigg, *C. gallinacea* 08-1274/3, *C. avium* 10DC88, *C. felis* Fe/C-56, *C. caviae* GPIC, *C.* sp. S15-834C (poikilothermis), *C.* sp. 2742-308 (sanzinia), *C.* sp. H15-1957-10C (serpentis). b) Species phylogenetic tree constructed based on gene order, the lengths of tree branches correspond to gene order similarity. (PDF 14 kb)

Additional file 14 Phylogenetic tree of genus *Chlamydia* with outgroup *Waddlia chondrophila*. The tree was constructed based on concatenated nucleotide sequence of 302 genes shared by all 227 strains of genus *Chlamydia* and *Waddlia chondrophila* WSU 86-1044 taken as outgroup to root the tree. Only single copy genes without frameshift and nonsense mutations were considered. Bootstrap values are shown below the edges as percentages. Branch lengths are ignored for readability, and actual values are shown above the edges. (PDF 2275 kb)

Additional file 15 Distribution of OGs with mosaic phyletic patterns across species (complete genomes only). (CSV 7 kb)

Additional file 16 Distribution of OGs with mosaic phyletic patterns across species (all genomes). (CSV 10 kb)

Additional file 17 Summary of peripheral genes’ phyletic patterns and tree concordance. (CSV 26 kb)

Additional file 18 Orthologous families (OFs) derived by MCL clustering of OGs. (CSV 189 kb)

Additional file 19 Additional information on proteins from OG1. (CSV 30 kb)

Additional file 20 Frameshift and nonsense mutations near homopolymeric tracts of OG1 genes. Only 374 genes with typical length and domain composition were considered. (CSV 6 kb)

Additional file 21 All polyN tracts of length 5 or more nucleotides in sequences of genes from OG1. Sequences were extracted and scanned prior to automatic correction for frameshifts implemented in the RAST pipeline. (CSV 133 kb)

## Data Availability

All datasets on which the conclusions of the paper rely presented in the main manuscript and additional supporting files. Additionally the table with complete information about all protein sequences used in the analysis can be provided upon request.

## Abbreviations

COG: Cluster of orthologous genes
GO terms: Gene ontology
HPT: Hexose phosphate transporter
Inc: Inclusion membrane protein
IS: Insertion element
LGV: Lymphogranuloma venereum
OF: Orthologous family
OG: Orthologous group
PLD: Phospholipase D
PmpG: Polymorphic membrane protein G
PZ: Plasticity zone
TCA: Tricarboxylic acid cycle
TF: Transcription factor
