# Supplementary figures and images for: Chlamydia pan-genomic analysis reveals balance between host adaptation and selective pressure to genome reduction
Source: BMC Genomics. 2019 Sep 12;20:710. doi: 10.1186/s12864-019-6059-5 (PMC6740158; doi:10.1186/s12864-019-6059-5)

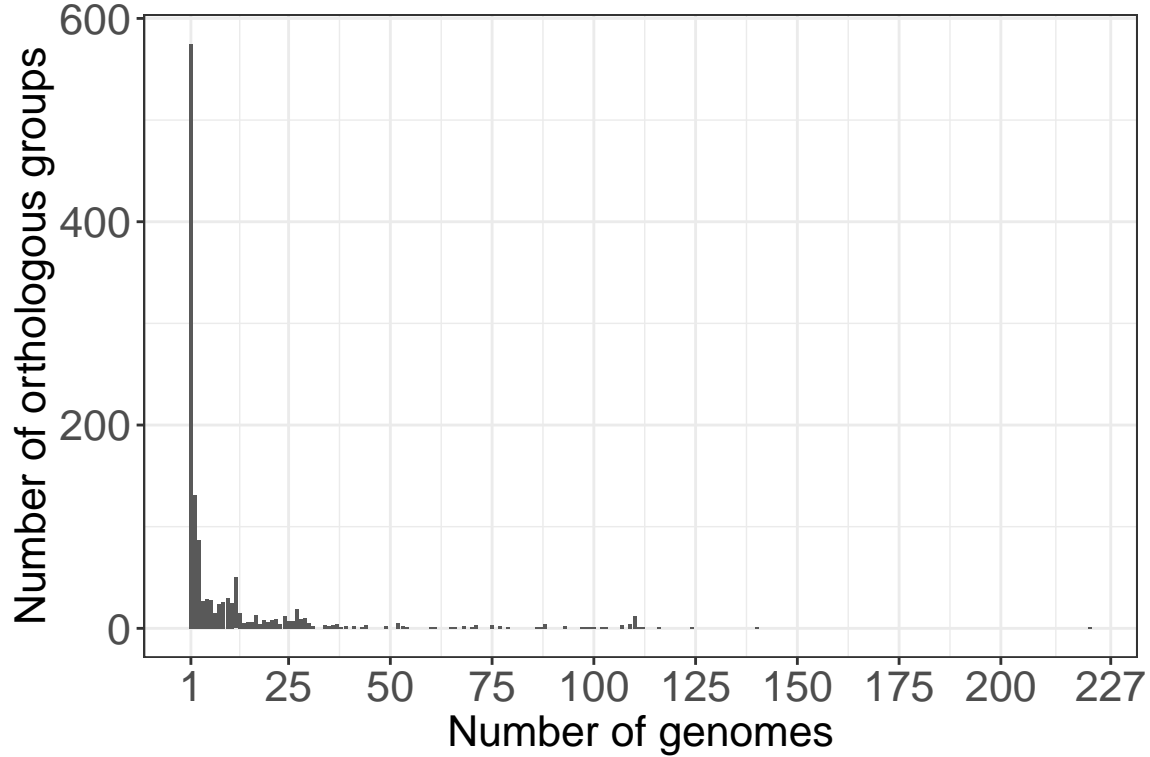

Supplement: Supplementary file 4 — Histogram of 1288 orthologous groups containing only hypothetical proteins with the average length below 50 amino acids by the number of genomes that have these OGs. These OGs were removed as potential false positives of gene prediction algorithms. (PDF 5 kb) [file 12864_2019_6059_MOESM4_ESM.pdf]

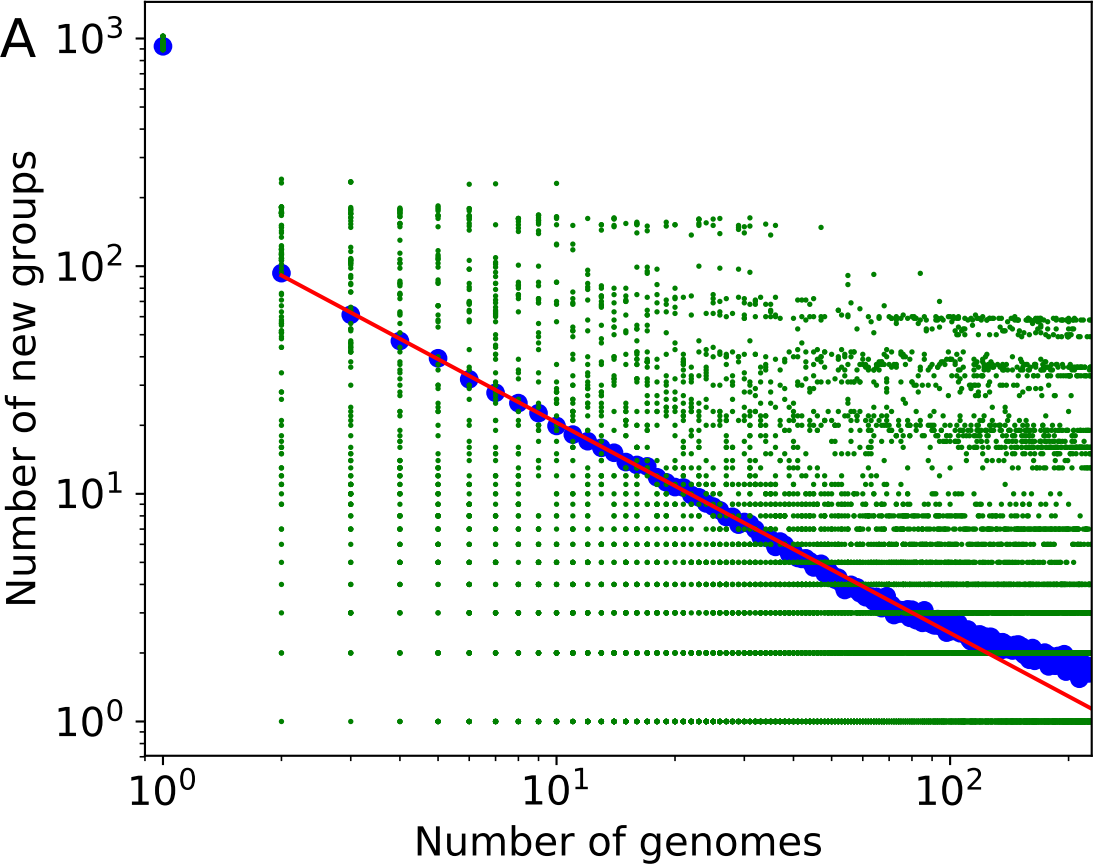

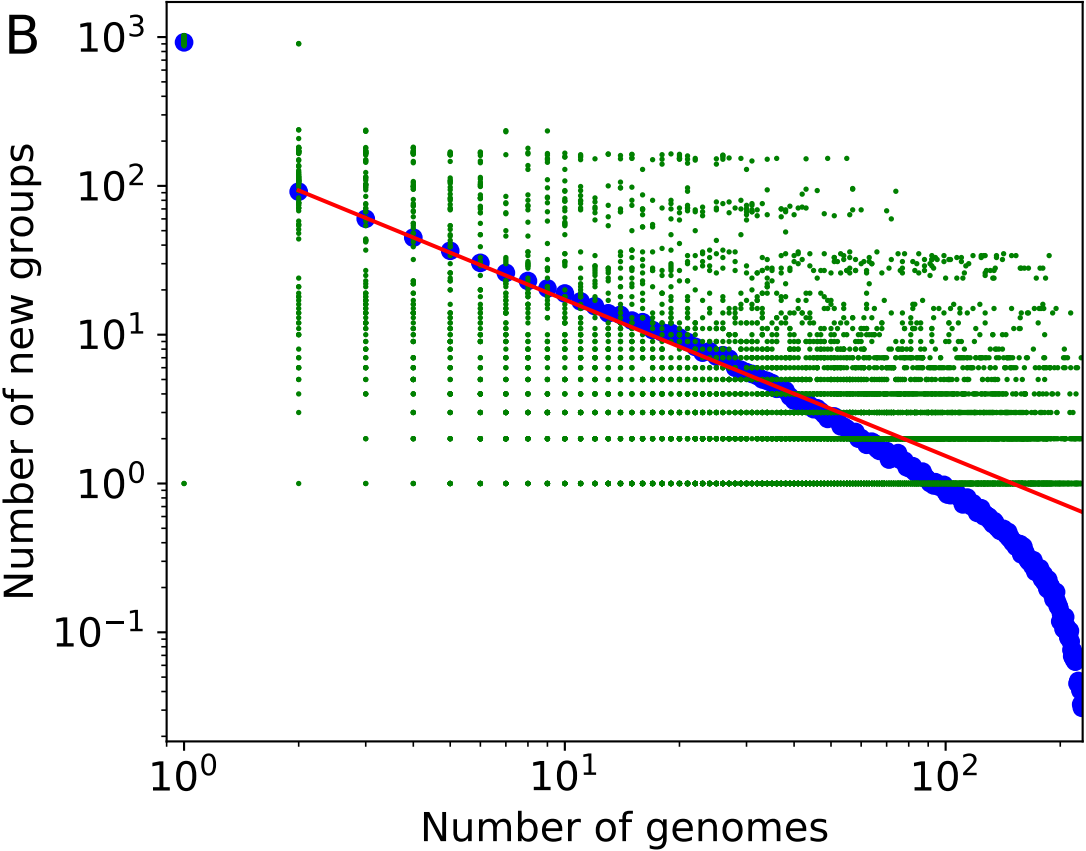

Supplement: Supplementary file 8 — The number of new genes added to the pan-genome upon addition of new strains of Chlamydia spp. The number of new genes is plotted as a function of the number (n) of strains sequentially added (see the model in [94]). For each n, points are the values obtained for different strain combinations; red symbols are the averages of these values. The superimposed line is the best fit with a decaying power law y=A·nξ. The pan-genome is considered open for ξ>−1 and converges to a constant for ξ<−1. (A) Full pan-genome, N(n)=173·n−0.92, (B) only OGs present in at least two genomes, N(n)=193·n−1.05. (CSV 7334 kb) [file 12864_2019_6059_MOESM8_ESM.pdf]

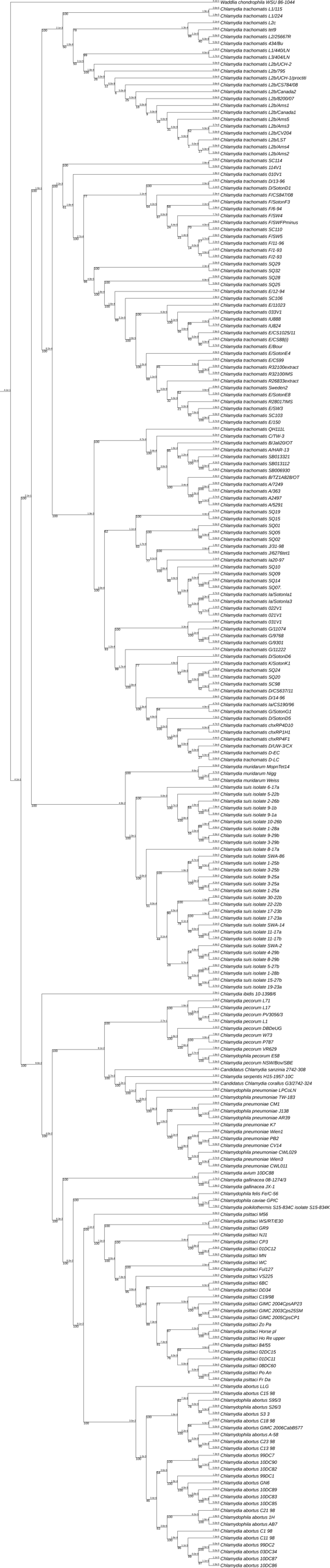

Supplement: Supplementary file 12 — Nonsynonymous to synonymous substitutions ratio (dN/dS) for the selected core OGs with a high number of frameshift and nonsense mutations (vertical red lines) compared to the overall dN/dS distribution for core genes (histogram). OG134: fumarate hydratase, class II, OG671: succinate dehydrogenase flavoprotein, OG670: succinate dehydrogenase iron-sulfur protein, OG458: arginine/ornithine antiporter, OG457: arginine decarboxylase, OG672: succinate dehydrogenase cytochrome b558 subunit. (PDF 47 kb) [file 12864_2019_6059_MOESM12_ESM.pdf]

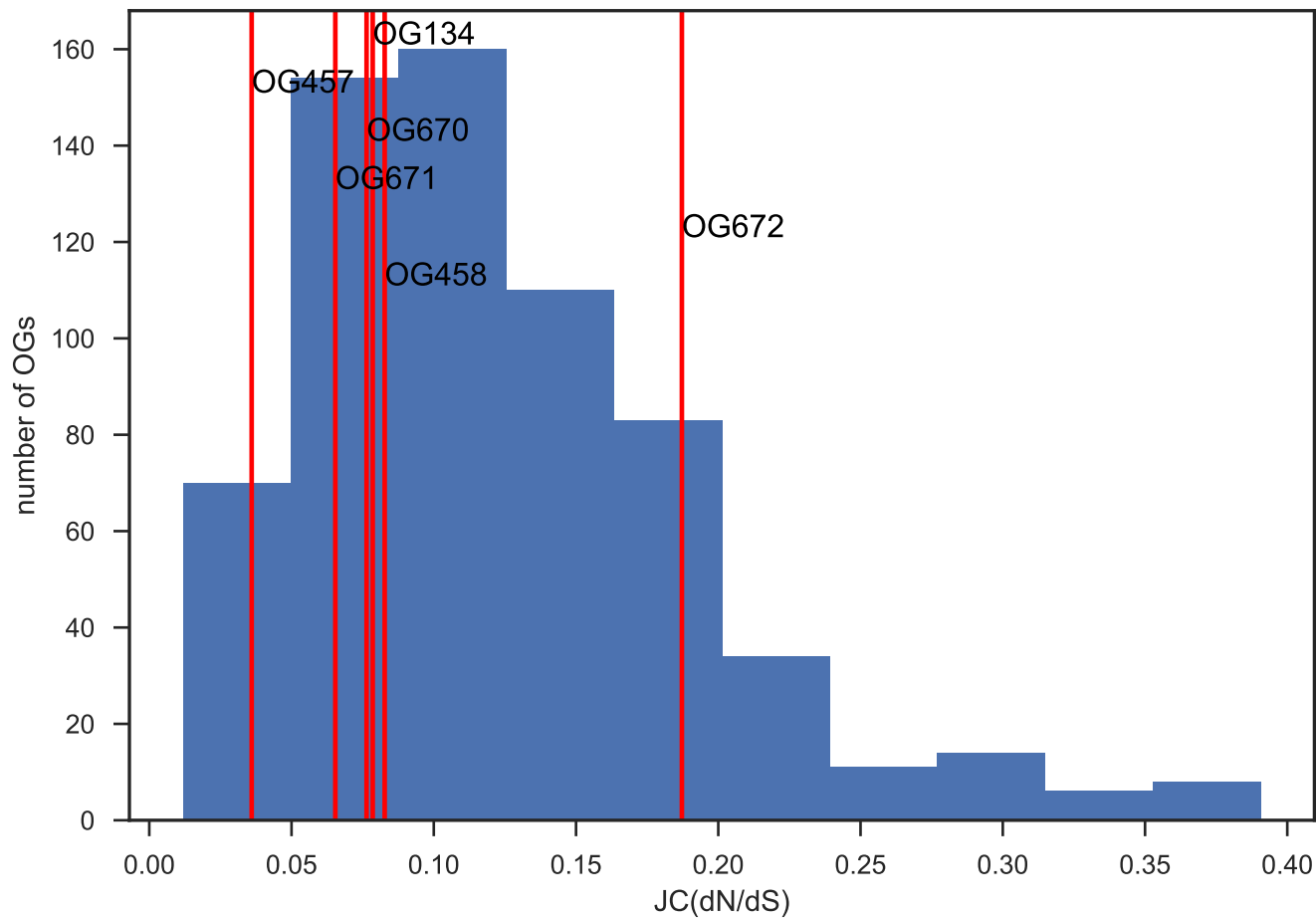

Supplement: Supplementary file 13 — Rearrangements in Chlamydia genomes. a)Full-genome alignment of Chlamydia genomes. Reference strains used for synteny blocks construction are C. trachomatis D/UW-3/CX, C. suis SWA-2, C. abortus S26/3, C. psittaci 6BC, C. pneumoniae CWL029, C. pecorum E58, C. muridarum Nigg, C. gallinacea 08-1274/3, C. avium 10DC88, C. felis Fe/C-56, C. caviae GPIC, C. sp. S15-834C (poikilothermis), C. sp. 2742-308 (sanzinia), C. sp. H15-1957-10C (serpentis). b) Species phylogenetic tree constructed based on gene order, the lengths of tree branches correspond to gene order similarity. (PDF 14 kb) [file 12864_2019_6059_MOESM13_ESM.pdf]

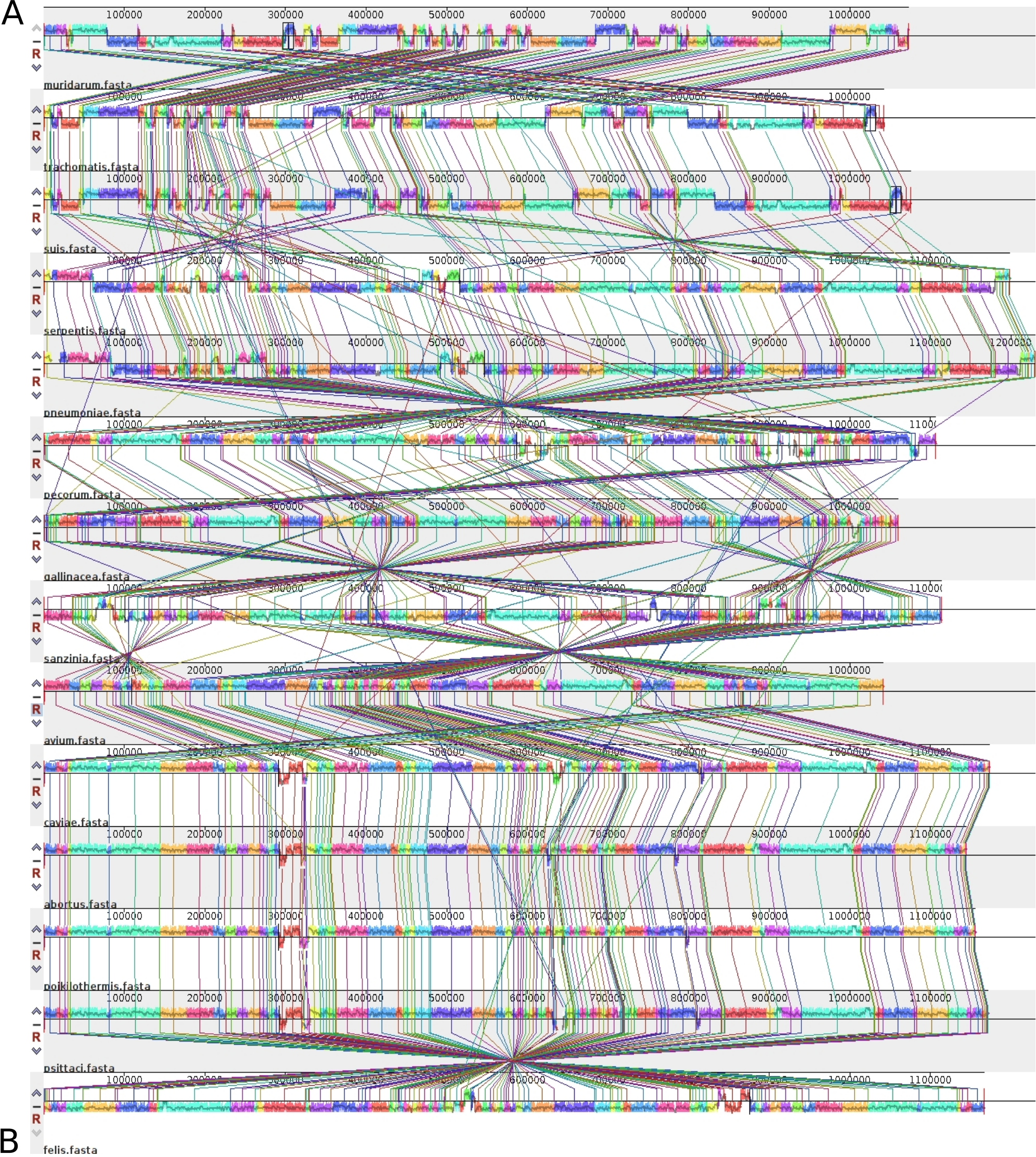

Supplement: Supplementary file 14 — Phylogenetic tree of genus Chlamydia with outgroup Waddlia chondrophila. The tree was constructed based on concatenated nucleotide sequence of 302 genes shared by all 227 strains of genus Chlamydia and Waddlia chondrophila WSU 86-1044 taken as outgroup to root the tree. Only single copy genes without frameshift and nonsense mutations were considered. Bootstrap values are shown below the edges as percentages. Branch lengths are ignored for readability, and actual values are shown above the edges. (PDF 2275 kb) [file 12864_2019_6059_MOESM14_ESM.pdf]
