# Supplementary material for: Chlamydia pan-genomic analysis reveals balance between host adaptation and selective pressure to genome reduction
Source: BMC Genomics. 2019 Sep 12;20:710. doi: 10.1186/s12864-019-6059-5 (PMC6740158; doi:10.1186/s12864-019-6059-5)
Supplement: Supplementary file 6 — Distribution of orthologous groups by the number of strains that have them for seven Chlamydia species with more than two available genomes. (PDF 8 kb) [file 12864_2019_6059_MOESM6_ESM.pdf]

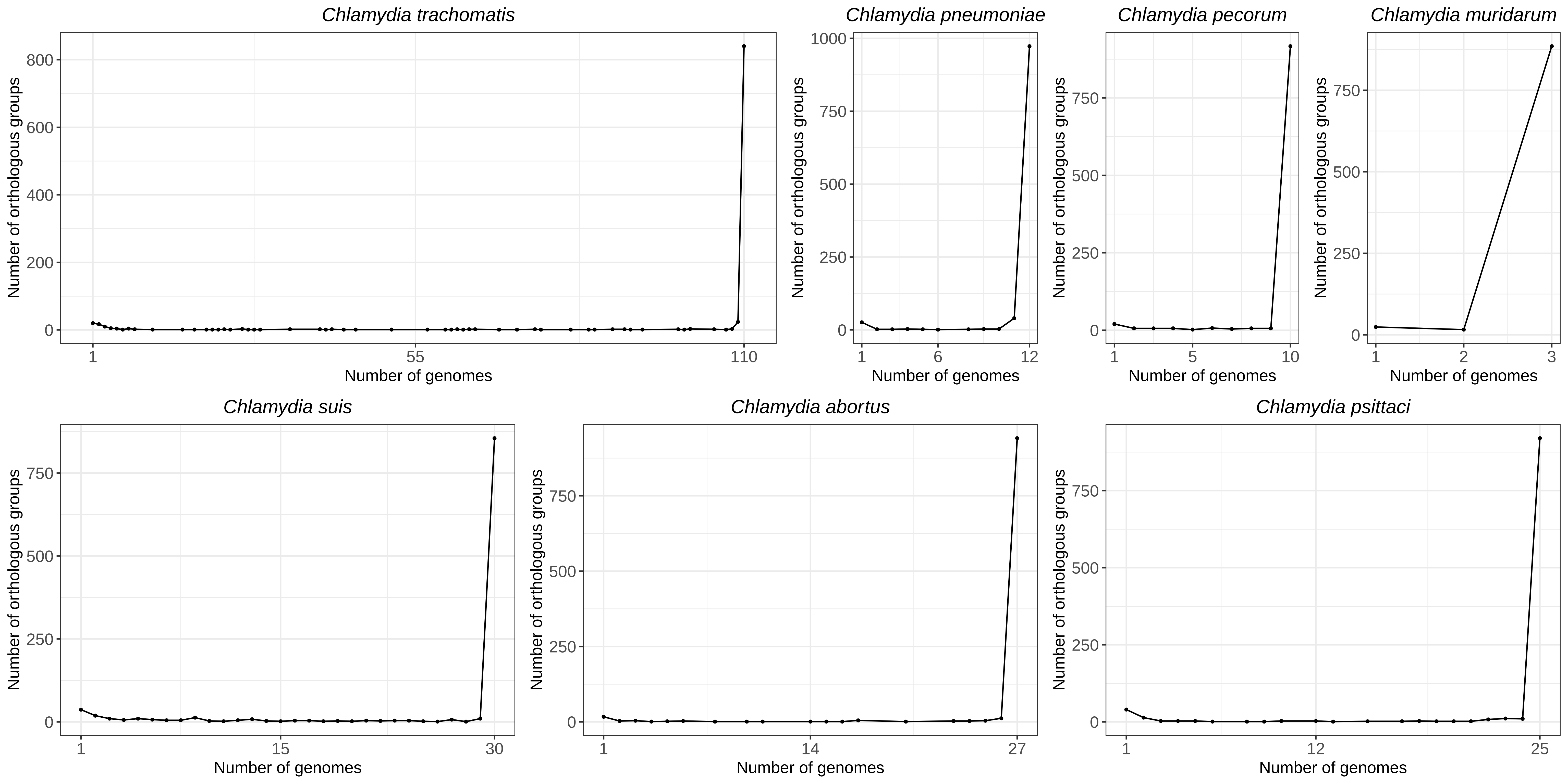

Chlamydia trachomatis
Chlamydia pneumoniae
Chlamydia pecorum
Chlamydia muridarum
Chlamydia suis
Chlamydia abortus
Chlamydia psittaci
Number of orthologous groups
Number of genomes
0
200
400
600
800
1
55
110
250
500
750
1000
6
12
5
10
2
3
15
30
14
27
12
25
