# Supplementary material for: Chlamydia pan-genomic analysis reveals balance between host adaptation and selective pressure to genome reduction
Source: BMC Genomics. 2019 Sep 12;20:710. doi: 10.1186/s12864-019-6059-5 (PMC6740158; doi:10.1186/s12864-019-6059-5)
Supplement: Supplementary file 7 — Distribution of orthologous groups by the number of strains that have them based on 161 complete genomes (red) or all 227 genomes assembled in at most 10 contigs (black). The pan-genome structure is not affected by genes potentially missing from 66 draft genomes. (PDF 7 kb) [file 12864_2019_6059_MOESM7_ESM.pdf]

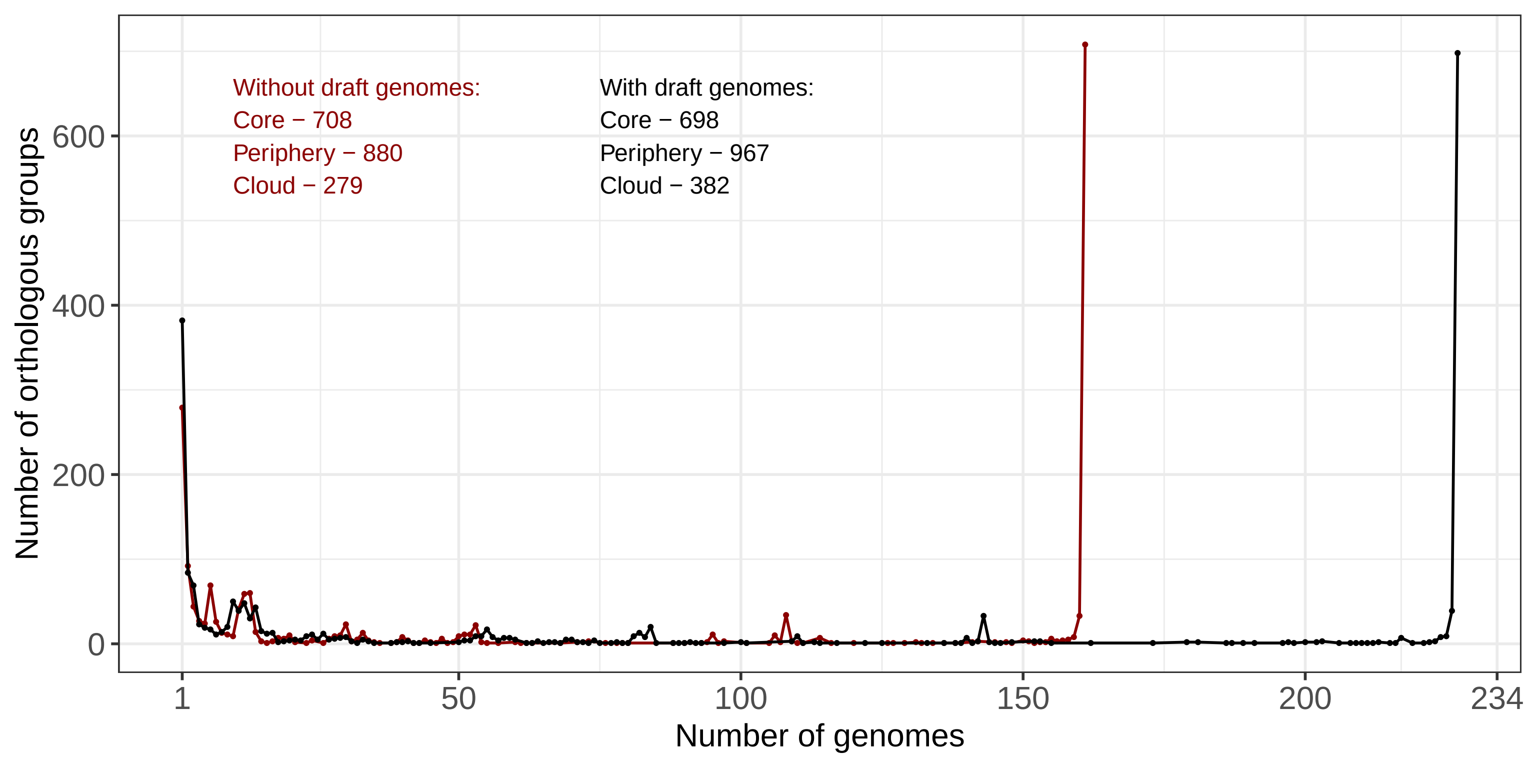
Without draft genomes:
Core − 708
Periphery − 880
Cloud − 279
With draft genomes:
Core − 698
Periphery − 967
Cloud − 382
Number of orthologous groups
0
200
400
600
1
50
100
150
200
234
Number of genomes
